# Analysis of Chemical Properties of Edible and Medicinal Ginger by Metabolomics Approach

**DOI:** 10.1155/2015/671058

**Published:** 2015-10-01

**Authors:** Ken Tanaka, Masanori Arita, Hiroaki Sakurai, Naoaki Ono, Yasuhiro Tezuka

**Affiliations:** ^1^College of Pharmaceutical Science, Ritsumeikan University, 1-1-1 Noji-Higashi, Kusatsu, Shiga 525-8577, Japan; ^2^Center for Information Biology, National Institute of Genetics, Mishima, Shizuoka 411-8540, Japan; ^3^RIKEN Center for Sustainable Resource Science, Yokohama, Tsurumi 230-0045, Japan; ^4^Department of Cancer Cell Biology, Graduate School of Medicine and Pharmaceutical Sciences, University of Toyama, Toyama 930-0194, Japan; ^5^Graduate School of Information Science, Nara Institute of Science and Technology, Ikoma, Nara 630-0192, Japan; ^6^Faculty of Pharmaceutical Sciences, Hokuriku University, Ho-3 Kanagawa-machi, Kanazawa 920-1181, Japan

## Abstract

In traditional herbal medicine, comprehensive understanding of bioactive constituent is important in order to analyze its true medicinal function. We investigated the chemical properties of medicinal and edible ginger cultivars using a liquid-chromatography mass spectrometry (LC-MS) approach. Our PCA results indicate the importance of acetylated derivatives of gingerol, not gingerol or shogaol, as the medicinal indicator. A newly developed ginger cultivar, *Z. officinale* cv. Ogawa Umare or “Ogawa Umare” (OG), contains more active ingredients, showing properties as a new resource for the production of herbal medicines derived from ginger in terms of its chemical constituents and rhizome yield.

## 1. Introduction

Traditional medicine embodies accumulation of knowledge, skills, and practices on the maintenance of health as well as the prevention, diagnosis, improvement, or treatment of physical and mental illness. World Health Organization reported that, even today, more than 80% of the world's population utilizes traditional medicine for primary health care [1]. Such medicinal system prescribes a combination of herbal, animal, and mineral parts, collectively known as crude drug, whose core materials are derived from plants including seeds, berries, roots, leaves, bark, or flowers [2]. The chemical constituents of crude drug are therefore considered a “chemical system,” which consists of a complex mixture of primary and secondary metabolites such as saponins, flavonoids, and alkaloids. The system is represented as a matrix in which rows and columns represent natural species and their chemical ingredients, respectively. This matrix works on another matrix representing the human body system, in which rows and columns represent interactive biomolecules (e.g., genes, proteins, and metabolites) and their tissue distribution, respectively. Thus, the research on traditional medicine deals with this “system to system” methodology, instead of the “point to point” methodology of western medicines (e.g., one particular chemical and its receptor gene). To understand the total function of traditional medicine, the knowledge of the interactions between matrices representing “chemical system” and “body system” is crucial. The matrix representing the human body system has gradually been made clear through several omics approaches, whereas knowledge on chemical system is not enough since almost all studies were done based on “point to point” or “point to system” methodology. Thus, we are accumulating the knowledge on chemical system with metabolomics approach [3–6].

Following our previous report on a newly registered turmeric (*Curcuma longa* cv. Okinawa Ougon) [3], we recently here investigate the chemical system of ginger cultivars. Ginger, the rhizome of the plant* Zingiber officinale *Roscoe, is widely used as a spice and herbal medicine for the treatment of catarrh, rheumatism, nervous diseases, gingivitis, toothache, asthma, stroke, constipation, and diabetes [7]. The genus* Zingiber* is distributed in tropical and subtropical Asia, Far East Asia, and Africa and is under cultivation mostly in India and China. The global consumption of ginger has been increasing rapidly, and the recent, growing demand for natural products as additives for functional food and beverages makes ginger an ideal candidate for development. Thus, attempts at crop improvement for ginger have been performed in order to increase the yield and enhance the concentration of its active constituents. Traditionally, crop improvement involves controlled crosses (hybridization) between selected cultivars with desirable properties.

The target of our metabolomic approach is three medicinal ginger types, “Shokyo” (dried rhizome of* Z. officinale* var.* rubens*), “Kankyo” (steamed and dried rhizome of* Z. officinale* var.* rubens*) from Kampo (traditional Japanese) medicine, and “Red ginger” (rhizome of* Z. officinale *var.* rubra*) from Indonesian traditional medicine (Jamu) [8, 9], and two edible ginger types, “Shoga” (fresh rhizome of* Z. officinale* var.* rubens*) from Japan and “White ginger” (rhizome of* Z. officinale* var.* amarum*) from Indonesia. From the comparison of five cultivars, we evaluate a new cultivar,* Z. officinale* cv. Ogawa Umare or “Ogawa Umare” (OG), and show its effectiveness as crude drug. OG was recently registered in the Japanese Plant Variety Protection (Ministry of Agriculture, Forestry and Fisheries, Japan) [10] and is characterized by its bold rhizome (3 times bigger than ordinary ginger) and a more pungent taste than standard medicinal ginger. All assays were conducted in a metabolomics platform with LC-MS and our results are consistent with the ginger taste.

## 2. Experimental

### 2.1. Specimens

The specimens of OG and “Shoga” used in this study were obtained from an official breeder. Fresh rhizomes of OG and “Shoga” were sliced and air-dried. Two specimens of Indonesian ginger, “Red ginger” and “White ginger,” were purchased from Oryza Oil & the Fat Chemical Co., Ltd. (Nagoya, Japan). Two Japanese herbal medicines, “Shokyo” and “Kankyo,” were bought from Tochimoto Tenkaido (Osaka, Japan). All specimens were deposited in the Museum of Materia Medica, College of Pharmaceutical Science, Ritsumeikan University (RIN).

### 2.2. Analytical Instruments

LC-MS analyses were performed using a Shimadzu LC-IT-TOF mass spectrometer equipped with an ESI interface. The ESI parameters were as follows: source voltage 4.5 kV, capillary temperature 200°C, and nebulizer gas 1.5 L/min. The LC-MS mass spectrometer was operated in the negative ion mode, scanning from* m/z* 50 to 2000. In the LC-MS analysis, a Waters Atlantis T3 column (2.1 mm i.d. × 150 mm) was used and the column temperature was maintained at 40°C. The mobile phase was a binary eluent of (A) 0.1% HCOOH solution and (B) CH_3_CN under the following gradient conditions: 0–30 min linear gradient from 20% to 100% B, 30–40 min isocratic maintained at 100% B. The flow rate was 0.2 mL/min.

### 2.3. LC-MS Sample Preparation

Individual specimens were homogenized to a fine powder using a multibeads shocker (Model MB755U, Yasui Kikai Co., Osaka, Japan). Two grams of the fine powder was accurately weighted and extracted four times with 50 mL of methanol under reflux conditions for 30 min. After centrifugation, the methanol layers were combined and evaporated* in vacuo* to give an extract. The extract was dissolved in 10 mL of methanol and filtrated through 0.2 *μ*m Millipore filter (polytetrafluoroethylene (PTFE) filter). Two milliliters of this solution was injected into LC-MS.

### 2.4. Standard Samples and Reagents

The isolated compounds ([6]-gingerol, [6]-shogaol, [6]-gingerdiol, and diacetoxy-[6]-gingerdiol) were identified by comparing their ^1^H- and ^13^C-NMR spectra with those reported in the literature [11, 12]. All chemicals were of analytical grade, and chromatographic solvents were of HPLC grade.

### 2.5. Data Analysis

All statistical analyses were carried out using Pirouette software (GL Science Inc., Tokyo).

### 2.6. Cell Proliferation Assay

HT-29 human colon cancer cells were seeded in 96-well plates (1 × 10^3^ cells/well). Cells were allowed to adhere to overnight culture and then treated with metabolites at the final concentration of 3–100 *μ*M. After a 72 h incubation, cell viability was determined with a WST-1 reagent (DOJINDO, Kumamoto, Japan).

## 3. Results and Discussion

**Figure 1:**
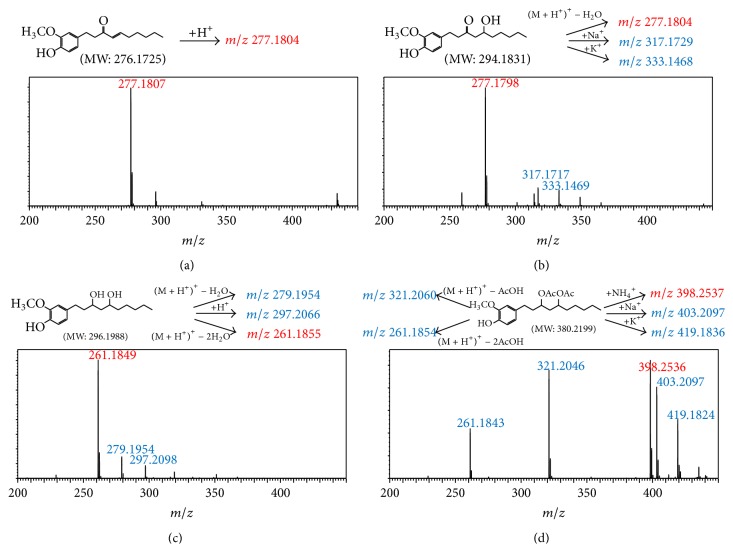
Mass spectra of (a) [6]-shogaol, (b) [6]-gingerol, (c) [6]-gingerdiol, and (d) diacetoxy-[6]-gingerdiol.

The major pungent principles of ginger are gingerols and shogaols (dehydrated form of gingerols). The conversion of gingerols to shogaols is favored at higher temperature [7], and shogaols show stronger activity than gingerols [13]. As Japanese “Shoga” contains lower amount shogaol than Chinese one, heat processing is used for the production of herbal medicines derived from ginger. In this study, the oleoresins and their derivatives such as gingerdiols, acetoxy gingerdiols, and diacetoxy gingerdiols [7] were identified based on mass spectral fragmentations with high-resolution mass data (Table 1). The fragmentation processes for [6]-gingerol, [6]-shogaol, [6]-gingerdiol, and diacetoxy-[6]-gingerdiol were determined from their mass spectra shown in Figure 1. [6]-Shogaol gave the (M+H)^+^ ion at* m/z* 277.1807, whereas [6]-gingerol did not provide the (M+H)^+^ ion and showed (M+Na)^+^ and (M+K)^+^ ions at* m/z* 317.1717 and 333.1469, respectively, together with [(M+H)–H_2_O]^+^ ion at* m/z* 277.1798. [6]-Gingerdiol predominantly provided the [(M+H)–2H_2_O]^+^ ion at* m/z* 261.1849 together with the weak (M+H)^+^ and [(M+H)–H_2_O]^+^ ions. In the case of diacetoxy-[6]-gingerdiol, intense signals for the [(M+H)–CH_3_COOH]^+^ and [(M+H)–2CH_3_COOH]^+^ ions were observed at* m/z* 321.2046 and 261.1843, respectively. Furthermore, three characteristic adduct ions, (M+NH_4_)^+^, (M+Na)^+^, and (M+K)^+^, were detected. These results indicate that gingerol, shogaol, and their related compounds could be annotated by ESI mass spectral patterns together with high-resolution mass data.

**Figure 2:**
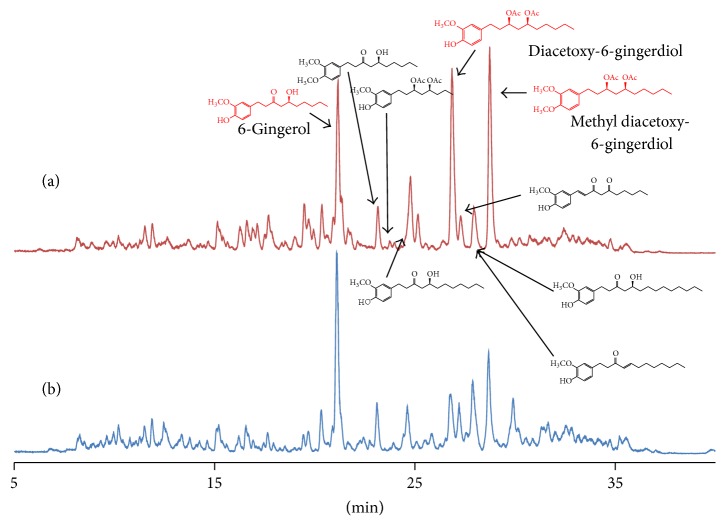
LC-MS chromatograms of (a) OG and (b) “Shoga.”

LC-MS chromatograms of OG and “Shoga” are shown in Figure 2. Intense peaks in the respective chromatogram were annotated by detailed analysis of their mass spectral data. Comparison of the chromatographic data shows that OG contains larger amounts of diacetoxy-[6]-gingerdiol and methyl diacetoxy-[6]-gingerdiol than “Shoga.”

**Figure 3:**
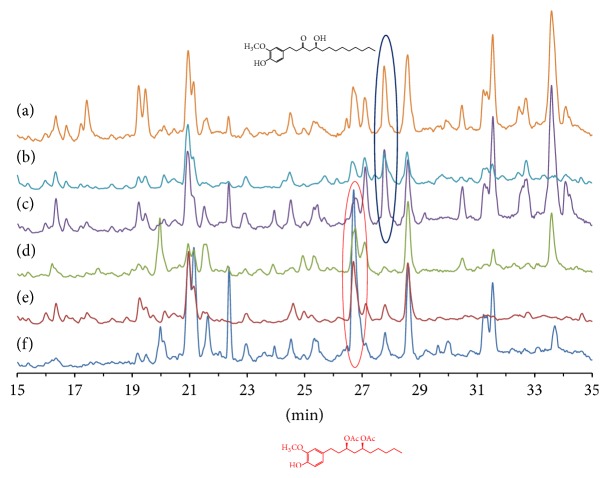
LC-MS chromatograms of the extracts of (a) dry ginger (“Shokyo”), (b) “Shoga” (*Z. officinale* var.* rubens*), (c) “White ginger” (*Z. officinale* var.* amarum*), (d) steamed and dried ginger (“Kankyo”), (e) “Ogawa Umare” (*Z. officinale* cv. Ogawa Umare, OG), and (f) “Red ginger” (*Z. officinale* var.* rubens*).

In order to clarify the medicinal properties of ginger, LC-MS chromatograms of the extracts of all six ginger types are shown in Figure 3. Although there are clear visual differences between the chromatograms of the upper three and lower three samples in Figure 3, this classification does not match their medicinal usage or tastes. For more unbiased interpretation and to reduce the dimensionality of the multivariate data, we analyzed the LC-MS chromatographic data using principal component analysis (PCA).

**Figure 4:**
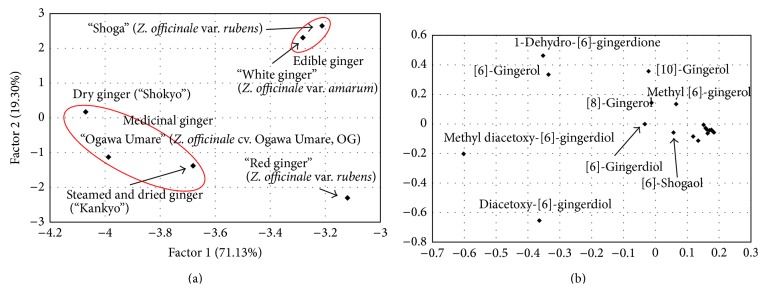
The score plot (a) and the loading plot (b) of PCA.

**Figure 5:**
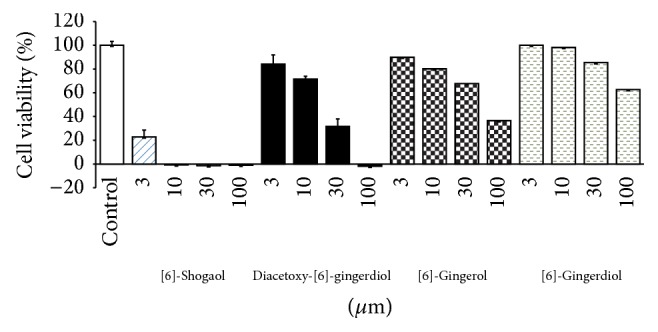
Cell viability of HT-29 human colon cancer cells after treatment with [6]-shogaol, diacetoxy-[6]-gingerdiol, [6]-gingerol, and [6]-gingerdiol.

PCA is an unsupervised method of multivariate data analysis and is used for clarifying the characteristic properties of the metabolomic profiles of complex mixtures, such as plant extracts. The annotated peaks and relative intensities detected in the chromatograms of the extracts (Table 2) were normalized and subjected to the PCA analysis. In Figure 4, the PCA scores plot and loading plot were shown. The first two PCs accounted for 90.4% of total variance (PC1, 71.1%; PC2, 19.3%). The scores plot clearly indicated that the chemical content patterns of the medicinal and edible ginger were different. In the chemometric analysis, the peaks having big loading values could be considered as the makers strongly contributing to the classification of the samples by PCA. In the present results, “Shokyo” and “Kankyo” showed similar properties, which were higher concentrations of acetoxy-[6]-gingerdiol and diacetoxy-[6]-gingerdiol. “Red ginger” was also characterized by its higher content of acetylated compounds, but low methyl diacetoxy-[6]-gingerdiol content. The new cultivar, OG, was also grouped with medicinal ginger. On the other hand, two edible ginger types (raw* Z. officinale* var.* rubens* and* Z. officinale* var.* amarum*) showed higher contents of [10]-gingerol and lower contents of acetylated compounds. Although* Z. officinale* var.* rubens* is used as the raw material in the production of Kampo medicine (“Shokyo” and “Kankyo”), only the most pungent fresh ginger is selected and utilized [14], which suggests the importance of shogaols and gingerols, the pungent and active constituents, for medicinal purpose [8]. So far, [6]-gingerol and [6]-shogaol were described as main bioactive constituents of ginger with “point to point” methodology [15–17], whereas [6]-gingerol was reported to be metabolized to (3*R*,5*S*)- and (3*S*,5*S*)-6-gingerdiols in mice to induce cell death toward H-1299 cancer cells [11]. On the other hand, our metabolomics approach to chemical system of medicinal ginger is based on “system to system” methodology and has suggested the importance of acetylated compounds, diacetoxy-[6]-gingerdiol. Thus, we examined the cytotoxicity of diacetoxy-[6]-gingerdiol, a main constituent of OG. As shown in Figure 5, diacetoxy-[6]-gingerdiol exhibited stronger cytotoxicity to HT-29 human colon cancer cells than [6]-gingerol. These results should indicate the importance of acetylated compounds such as diacetoxy-[6]-gingerdiol for the use as Kampo medicine and for the classification of medicinal and edible ginger. In addition, from the viewpoint of its chemical constituents and rhizome yield, OG has valuable properties as a new resource for the production of herbal medicines derived from ginger.

## 4. Conclusion

Up to now, several studies reported on the contribution of [6]-gingerol and [6]-shogaol to many biological activities of ginger. Prasad and Tyagi summarized many molecular targets of the compounds [15]. However, medicinal activities of ginger are not attributable to only [6]-gingerol and [6]-shogaol. Their derivatives have been actively investigated for novel bioactivities such as antihaemolysis by longer chain oleoresins [18], quorum sensing inhibition by [6]-azashogaol [19], and antiplatelet aggregation by [6]-paradol [20]. Synergistic bioactivity of [6]-gingerol with another metabolite is also reported [21]. Our observation that acetoxy derivatives are relatively abundant in medicinal ginger and the compound possesses biological activities may provide additional clues to find more bioactivities of ginger. On the other hand, scarcity of [10]-gingerol, [12]-gingerol, or gingerdiols in both medicinal and edible ginger indicates that these bioactive components [22] play fewer roles in the medication of traditional medicines.

The molecular targets of the certain compounds have gradually been made clear through several omics approaches, whereas knowledge on chemical system is still limited. Integration of the knowledge of  “chemical system” as described in this paper may help understand the action between “chemical system” and “body system” in traditional medicines.

## Figures and Tables

**Table 1:** Compounds in ginger, their compositions, and expected weight of (M+H)^+^ ions.

Compounds	C	H	O	MW	[M+H]^+^	
[6]-Paradol	17	26	3	278.1882	279.1960	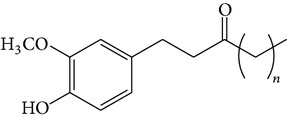
[7]-Paradol	18	28	3	292.2038	293.2117	
[8]-Paradol	19	30	3	306.2195	307.2273	
[9]-Paradol	20	32	3	320.2351	321.2430	
[10]-Paradol	21	34	3	334.2508	335.2586	
[11]-Paradol	22	36	3	348.2664	349.2743	
[13]-Paradol	24	40	3	376.2977	377.3056	
Methyl [6]-paradol	18	28	3	292.2038	293.2117	

[4]-Gingerol	15	22	4	266.1518	267.1596	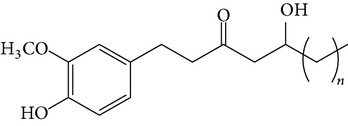
[6]-Gingerol	17	26	4	294.1831	295.1909	
[7]-Gingerol	18	28	4	308.1988	309.2066	
[8]-Gingerol	19	30	4	322.2144	323.2222	
[10]-Gingerol	21	34	4	350.2457	351.2535	
Methyl [4]-gingerol	16	24	4	280.1675	281.1753	
Methyl [6]-gingerol	18	28	4	308.1988	309.2066	

[4]-Shogaol	15	20	3	248.1412	249.1491	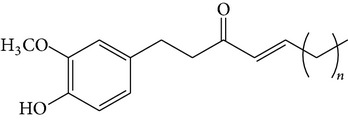
[6]-Shogaol	17	24	3	276.1725	277.1804	
[8]-Shogaol	19	28	3	304.2038	305.2117	
[10]-Shogaol	21	32	3	332.2351	333.2430	
[12]-Shogaol	23	36	3	360.2664	361.2743	
Methyl [6]-shogaol	18	26	3	290.1882	291.1960	
Methyl [8]-shogaol	20	30	3	318.2195	319.2273	

Acetoxy-[4]-gingerol	17	24	5	308.1624	309.1702	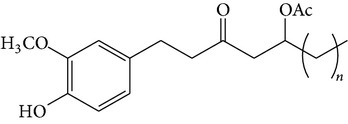
Acetoxy-[6]-gingerol	19	28	5	336.1937	337.2015	
Acetoxy-[8]-gingerol	21	32	5	364.2250	365.2328	
Acetoxy-[10]-gingerol	23	36	5	392.2563	393.2641	
Methyl acetoxy-[6]-gingerol	20	30	5	350.2093	351.2172	

1-Dehydro-[3]-gingerdione	14	16	4	248.1049	249.1127	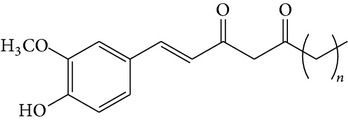
1-Dehydro-[6]-gingerdione	17	22	4	290.1518	291.1596	
1-Dehydro-[8]-gingerdione	19	26	4	318.1831	319.1909	
1-Dehydro-[10]-gingerdione	21	30	4	346.2144	347.2222	

[4]-Gingerdiol	15	24	4	268.1675	269.1753	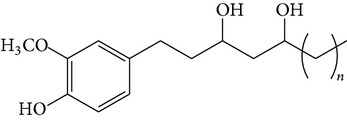
[6]-Gingerdiol	17	28	4	296.1988	297.2066	
[8]-Gingerdiol	19	32	4	324.2301	325.2379	
[10]-Gingerdiol	21	36	4	352.2614	353.2692	

5-Acetoxy-[4]-gingerdiol	17	26	5	310.1780	311.1859	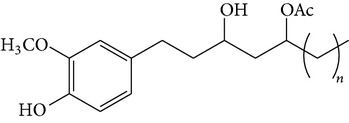
5-Acetoxy-[6]-gingerdiol	19	30	5	338.2093	339.2172	
5-Acetoxy-[7]-gingerdiol	20	32	5	352.2250	353.2328	
Methyl 5-acetoxy-[4]-gingerdiol	18	28	5	324.1937	325.2015	
Methyl 5-acetoxy-[6]-gingerdiol	20	32	5	352.2250	353.2328	

Diacetoxy-[4]-gingerdiol	19	28	6	352.1886	353.1964	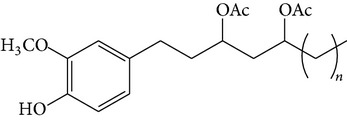
Diacetoxy-[6]-gingerdiol	21	32	6	380.2199	381.2277	
Methyl diacetoxy-[4]-gingerdiol	20	30	6	366.2042	367.2121	
Methyl diacetoxy-[6]-gingerdiol	22	34	6	394.2355	395.2434	
Methyl diacetoxy-[10]-gingerdiol	26	42	6	450.2981	451.3060	

**Table 2:** Annotated peaks and relative intensities detected in the chromatograms of the ginger extracts.

Compounds	Retention time (min)	Relative intensity					
		Red ginger	OG	Kankyo	White ginger	Shoga	Shokyo
[6]-Gingerdiol	20.13	63181888	34195478	35356476	29468319	38266151	40782586
[6]-Gingerol	20.93	167039972	46057215	20037041	163492434	78274022	136439541
Methyl [6]-gingerol	22.97	9115912	7474131	10056226	40450873	31652131	30072261
5-Acetoxy-[6]-gingerdiol	22.97	41701072	14320787	8371635	2022737	1469088	10809735
Diacetoxy-[4]-gingerdiol	23.58	0	3764215	3927640	773810	0	4670940
[8]-Gingerdiol	23.78	14502585	2161811	1983957	4419134	3260478	5932284
[8]-Gingerol	24.48	53406657	13470644	3991218	76799848	26551957	63339810
Acetoxy-[6]-gingerol	24.60	0	5066601	2597449	0	3259442	5569505
Methyl 5-acetoxy-[6]-Gingerdiol	24.95	13575229	17357866	18372059	5087324	3214607	18486380
[6]-Shogaol	25.37	55671256	0	34992211	44191998	2677276	33490436
Methyl [6]-shogaol	26.33	11794505	1309059	1624192	1752366	0	1482872
Methyl acetoxy-[6]-gingerol	26.33	17107183	7053201	5050693	4379036	1820760	3071943
Diacetoxy-[6]-gingerdiol	26.70	368541323	117403970	88966249	47412894	4212568	96827979
1-Dehydro-[6]-gingerdione	27.13	9896109	80710864	63993719	131863825	109603237	113232143
[10]-Gingerdiol	27.21	18056089	1755611	1728210	10924634	4379361	10721515
[10]-Gingerol	27.79	31802196	19048130	5682838	140215493	45855844	27.825
[8]-Shogaol	28.62	20999783	0	7432153	20607889	0	0
Methyl diacetoxy-[6]-gingerdiol	28.62	190863115	136670779	166419472	114138767	64094728	164358531
[10]-Shogaol	31.52	7237938	0	4268883	24167568	0	16005930
